# Development of a Fragment-Based Screening Assay for the Focal Adhesion Targeting Domain Using SPR and NMR

**DOI:** 10.3390/molecules24183352

**Published:** 2019-09-14

**Authors:** Carlos Alvarado, Erik Stahl, Karissa Koessel, Andrew Rivera, Brian R. Cherry, Surya V.S.R.K. Pulavarti, Thomas Szyperski, William Cance, Timothy Marlowe

**Affiliations:** 1 University of Arizona Cancer Center, 625 N. 6th Street, Phoenix, AZ 85004, USAestahl@email.arizona.edu (E.S.); arivera7793@gmail.com (A.R.); wcance@email.arizona.edu (W.C.); 2 University of Arizona College of Pharmacy-Phoenix, 650 E. Van Buren Street, Phoenix, AZ 85004, USA; koessel@pharmacy.arizona.edu; 3 Molecular Discovery Core, University of Arizona College of Medicine-Phoenix, 475 N. 5th Street, Phoenix, AZ 85004, USA; 4 The Magnetic Resonance Research Center, Arizona State University, Tempe, AZ 85287, USA; Brian.R.Cherry@asu.edu; 5 Department of Chemistry, State University of New York at Buffalo, Buffalo, NY 14260, USAszypersk@buffalo.edu (T.S.); 6 Interdisciplinary Oncology, University of Arizona College of Medicine-Phoenix, 475 N. 5th Street, Phoenix, AZ 85004, USA; 7 Pharmacology and Toxicology, University of Arizona College of Medicine-Phoenix, P.O. Box 210207, Tucson, AZ 85721, USA

**Keywords:** focal adhesion kinase, FAT domain, fragment-based drug discovery, surface plasmon resonance, nuclear magnetic resonance

## Abstract

The Focal Adhesion Targeting (FAT) domain of Focal Adhesion Kinase (FAK) is a promising drug target since FAK is overexpressed in many malignancies and promotes cancer cell metastasis. The FAT domain serves as a scaffolding protein, and its interaction with the protein paxillin localizes FAK to focal adhesions. Various studies have highlighted the importance of FAT-paxillin binding in tumor growth, cell invasion, and metastasis. Targeting this interaction through high-throughput screening (HTS) provides a challenge due to the large and complex binding interface. In this report, we describe a novel approach to targeting FAT through fragment-based drug discovery (FBDD). We developed two fragment-based screening assays—a primary SPR assay and a secondary heteronuclear single quantum coherence nuclear magnetic resonance (HSQC-NMR) assay. For SPR, we designed an AviTag construct, optimized SPR buffer conditions, and created mutant controls. For NMR, resonance backbone assignments of the human FAT domain were obtained for the HSQC assay. A 189-compound fragment library from Enamine was screened through our primary SPR assay to demonstrate the feasibility of a FAT-FBDD pipeline, with 19 initial hit compounds. A final total of 11 validated hits were identified after secondary screening on NMR. This screening pipeline is the first FBDD screen of the FAT domain reported and represents a valid method for further drug discovery efforts on this difficult target.

## 1. Introduction

Focal Adhesion Kinase (FAK) is a 125 kDa non-receptor tyrosine kinase overexpressed at the protein and mRNA levels in over 80% of solid malignant tumors [1,2,3]. Numerous studies have provided validation that FAK is critical for human cancer progression due to its role in cellular adhesion, motility, invasion, metastasis, and angiocrine signaling [4,5,6,7]. Knockdown studies of FAK in transgenic mouse models has shown decrease in tumor growth, invasion, and metastasis [8,9,10,11]. Inhibition of FAK through cellular expression of a C-terminal domain FAK construct results in loss of adhesion and induction of apoptosis on tumor cells [12]. FAK is composed of three primary domains: the N-terminal 4.1 ezrin, radizin, moesin (FERM) domain, the central kinase domain, and the C-terminal focal adhesion targeting (FAT) domain. The FERM and FAT domains allow FAK to function as a scaffolding protein in conjunction with its kinase activity [13,14]. Previous studies have shown that FAK-kinase inhibitors provide limited inhibition of FAK autophosphorylation at key residue Y397 due to drug resistance mechanisms involving Receptor Tyrosine Kinase (RTK) transphosphorylation of FAK [15]. Although inhibitors of the FAK-kinase domain have entered clinical trials [16,17], early results suggest limited efficacy.

An alternative drug-targeting approach is focusing on the FAT domain, a four-helical bundle, which has been shown to be involved in multiple protein-protein interactions at the focal adhesion site [18]. FAK localization to the focal adhesion is controlled through the FAT-Paxillin interaction [19]. Furthermore, mutation of the FAT-Paxillin binding site was shown to have significant effects on both FAK and Paxillin phosphorylation, cell adhesion, focal adhesion turnover, invasion, and metastasis [20,21,22]. Paxillin binds to FAT through alpha helical LD binding motifs named after the first two residues of the consensus sequence (LDXLLXXL). In particular, the LD2 motif (NLSELDRLLLELN) has been shown to bind to two different sites, one on the helix 1-4 interface and the other on the helix 2-3 interface [22]. Validation of the FAT-Paxillin interaction has been shown through X-ray crystallography, isothermal titration calorimetry (ITC), and mutagenesis, providing a new target to inhibit FAK through a non-catalytic mechanism [23,24,25,26]. While FAT remains an attractive target for inhibiting focal adhesion kinase, it has long been considered “undruggable.” The major issue behind targeting this interaction, as well as many other protein-protein interactions (PPIs), is the expansive, shallow, and unique interface of the binding interaction. Discovering a chemical compound with the exact specific properties necessary to target the FAT-Paxillin interaction is difficult utilizing traditional high throughput screening (HTS). This drawback has left the FAT domain largely unexplored in drug discovery efforts.

Fragment-based drug discovery (FBDD) is an alternative approach that can be utilized in properly targeting PPIs [27,28,29], including the FAT-Paxillin binding interface. Fragments are relatively small molecular compounds that are organized into libraries based on the Rule of Three: a molecular weight of less than 300 Daltons, a LogP of less than three, no more than three hydrogen bond donors and acceptors, and no more than three rotatable bonds [30]. The properties of these fragments allow for the exploitation of a greater chemical space, with hits being tailored more specifically to the desired target [31]. The larger molecular compounds used in HTS, due to their size and complexity, do not provide the same complementarity to the binding pocket as seen with FBDD. The FAT-Paxillin binding site is a protein-protein interaction that is difficult to target due to its intricate surface, but fragments have the capability to bind to regions of that larger surface. In comparison to HTS, FBDD hits usually possess lower binding affinities but bind with greater efficiency per number of heavy atoms (ligand efficiency) [31]. These fragment hits are then analyzed through various orthogonal assays and have the potential to be expanded or linked into higher molecular weight compounds utilizing medicinal chemistry approaches.

In this paper, we report the development and optimization of a primary surface plasmon resonance (SPR) assay and a secondary heteronuclear single quantum coherence nuclear magnetic resonance (HSQC-NMR) assay for the detection of fragment binders to the FAT-Paxillin binding interface. We show the development of several FAT constructs, including two single mutants and a double mutant, as controls for the SPR assay. We show the optimization of the SPR assay by testing different buffers and their effect on LD2 binding. We also report backbone resonance assignments of the human FAT domain (S892–H1052) sequence for HSQC-NMR studies. Finally, we screen a pilot (189 compound) fragment library through SPR and validate the hits through HSQC-NMR with a final hit count of 11.

## 2. Results

### 2.1. Development of a Surface Plasmon Resonance Screening Assay for the FAT Fomain

**Figure 1:**
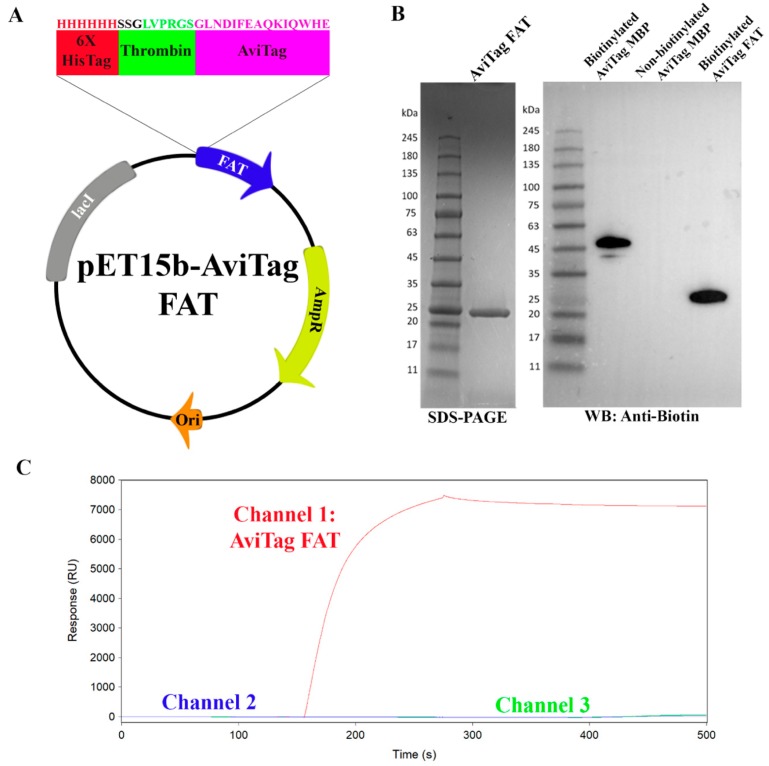
Construction and validation of AviTag Focal Adhesion Targeting (FAT). (**A**) Plasmid map of pET15b AviTag FAT with labeled protein sequence of the insert. The insert contains the 6x HisTag, 6 residue thrombin cleavage site, and the 15 residue AviTag sequence. (**B**) SDS-PAGE of purified AviTag FAT and western blot of biotinylated AviTag FAT probing with anti-biotin antibody. Biotinylated and non-biotinylated AviTag Maltose Binding Protein (MBP) were used as controls. The SDS-PAGE shows FAT purification at over 90% purity. The western blot shows a band for biotinylated AviTag FAT confirming successful biotinylation. (**C**) Surface plasmon resonance (SPR) sensorgram showing immobilization of biotinylated AviTag FAT to a SADH streptavidin coated chip. The final immobilization response level of this protein, shown in Channel 1, is at around 7000 RU. Channel 2 and Channel 3 do not have any immobilized protein and demonstrate a consistent baseline providing ideal reference channels.

Fragment-based screening requires very sensitive technologies to detect the weaker binding of fragments. Surface plasmon resonance has the capacity and sensitivity to detect such binding, making it a suitable platform to develop a fragment screening assay [32]. To develop a fragment-based screening assay for the FAT domain, we first started with optimization of the SPR immobilization technique. We avoided traditional amine coupling for immobilization, as this method would produce variable orientations of the protein on the SPR surface, given the FAT domain has multiple primary amines including a few key lysine residues in the helix 1-4 and 2-3 binding pockets [33]. As an alternative approach, we utilized the AviTag, a protein affinity tag that allows for site-specific biotinylation and uniform protein orientation on the surface of a streptavidin-coated chip [34]. Utilizing a previously established pET15b-FAT construct, an AviTag, a thrombin-cleavage site, and a hexa-histidine tag were cloned into the vector (Figure 1A). Subsequent purification and biotinylation were confirmed through sodium dodecyl-sulfate polyacrylamide gel electrophoresis (SDS-PAGE) and western blot, respectively (Figure 1B). Western blots of the biotinylated AviTag FAT construct demonstrated biotinylation efficiency levels comparable to commercially purchased biotinylated AviTag Maltose Binding Protein (MBP). Next, biotinylated AviTag FAT was tested for immobilization on a streptavidin coated SADH chip (Figure 1C). The protein immobilized to over 7000 RU, however only 7000 RU were necessary for the fragment studies in this paper as stated in the methods. Dissociation of AviTag FAT from the chip occurred at a low rate, with over half (3500 RU) still bound after three days.

### 2.2. Optimization of SPR Assay Buffer Conditions

**Figure 2:**
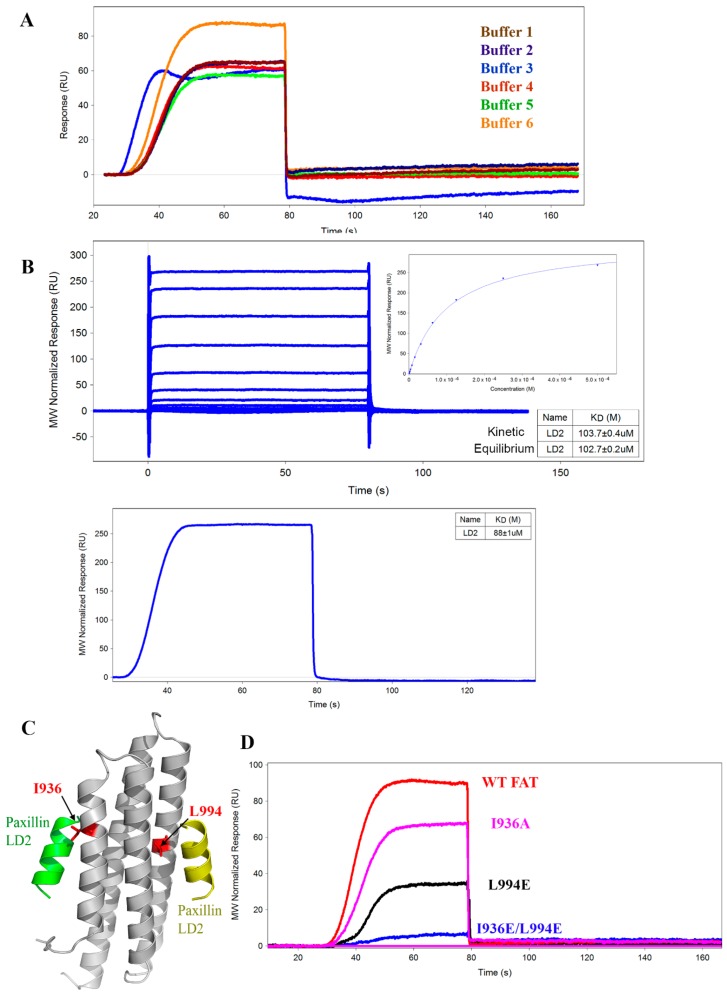
Development and optimization of SPR assay. (**A**) SPR sensorgram of AviTag FAT-LD2 binding in various buffers for optimization studies. The buffers listed are as follows-Buffer 1: 20 mM Tris-Cl pH 8.0, 200 mM NaCl, 1% DMSO; Buffer 2: 100 mM Tris-Cl pH 8.0, 200 mM NaCl, 1% DMSO; Buffer 3: 100 mM Tris-Cl pH 8.0, 200 mM NaCl, 1% DMSO, 0.05% Triton-X 100; Buffer 4: 100 mM Tris-Cl pH 8.0, 200 mM NaCl, 1% DMSO, 0.05% Tween-20; Buffer 5: 100 mM Tris-Cl pH 8.0, 200 mM NaCl, 2.5% DMSO, 0.05% Tween-20; Buffer 6: 100 mM Tris-Cl pH 8.0, 200 mM NaCl, 5% DMSO, 0.05% Tween-20. Buffer 6 demonstrated the largest response and best on-rate of all buffers examined and was chosen as the final buffer for all SPR runs moving forward. (**B**) Comparison between Paxillin-LD2 (50 µM) binding to wild-type (WT) AviTag FAT using a fixed concentration injection (FCI) (top) versus an OneStep injection (bottom). Kinetic fitting of FCI produced a K_D_ of 103.7 ± 0.4 µM and equilibrium fit produced a K_D_ of 102.7 ± 0.2 µM. OneStep injection produced a K_D_ of 88 ± 0.1 µM, very similar to the binding affinity by FCI. (**C**) Molecular model (PDB ID: IOW8) of the FAT-LD2 binding complex highlighting residues to be mutated as binding controls. (**D**) SPR sensorgram comparing the binding of LD2 against various FAT constructs, including WT FAT, single mutants I936A, L994E, and double mutant I936E/L994E. WT to mutant selectivity ratios were calculated from these results: I936A-1.5, L994E-3, I936E/L994E-18.

Buffer conditions are critical for SPR-based assays. Inadequate buffers can damage immobilized protein, promote analyte insolubility, and produce inaccurate responses. To optimize the buffer conditions, biotinylated AviTag FAT was immobilized onto a single channel on the SADH SPR chip, and LD2 (50 µM) binding to FAT was tested in various buffer conditions (Figure 2A). Buffer concentration (Tris; 20 vs. 100 mM), detergent type (Triton X-100 vs. Tween-20), and dimethyl sulfoxide (DMSO) concentration (1%, 2.5%, or 5%) were the variables examined through this study. The optimization results indicated that Tris concentration had no effect on binding, but 100 mM was ultimately chosen for the higher buffering capacity and ability to prevent any acid-base effects from the fragments. Triton X-100 in the buffer yielded binding curves indicative of non-specific interactions between immobilized protein and buffer, while Tween-20 avoided those effects. The highest DMSO concentration, 5%, displayed the highest R_eq_ at 80 RU, potentially attributable to an increased solubility of LD2. The optimal and final buffer chosen for all future studies was 100 mM Tris pH 8.0, 200 mM NaCl, 0.5% Tween-20, and 5% DMSO (Buffer 6).

### 2.3. Comparison of OneStep vs. Fixed Concentration Injection Kinetics

The FortéBio Pioneer FE SPR system has the unique OneStep injection feature that can calculate K_D_ from kinetic fitting of a single injection of an automatic concentration gradient [35]. Using this feature, this system can run an affinity-level screen of up to 768 analytes in 24 h and is therefore advantageous for fragment screening. To verify the accuracy of the OneStep technique in comparison to the standard fixed concentration injection (FCI) method, LD2 binding to AviTag FAT was measured through both methods (Figure 2B). For the standard FCI method, LD2 was titrated from 500 µM to 250 nM before injection into the system. Kinetic fitting of the data yielded a K_D_ of 103.7 ± 0.4 µM. Equilibrium fitting the data produced a binding isotherm with a K_D_ of 102.7 ± 0.2 µM for the LD2-FAT binding interaction. Subsequently, the OneStep method was run on a single sample of 500 µM LD2, and kinetic fitting of the resulting binding curve derived a K_D_ of 88 ± 0.1 µM. The resulting K_D_ were close in value, validating the OneStep protocol as a method for SPR screening moving forward. All following SPR runs were performed using the OneStep method.

### 2.4. Mutant AviTag FAT Constructs for SPR Assay Controls

To validate the specificity of the SPR signal, we created several mutant FAT proteins designed to prevent LD2 binding. Examining the X-ray crystal structure of the binding complex between Paxillin-LD2 and FAT (PDB ID: 1OW8), we selected two key interfacial residues (I936 and L994) involved in hydrophobic interactions necessary for LD2 binding to the helix 1-4 and helix 2-3 binding sites, respectively [36] (Figure 2C). Three different AviTag FAT mutant constructs, two single mutants (I936A, L994E) and one double mutant (L994E + I936E), were obtained. LD2 (50 µM) binding between wild-type FAT and all mutant proteins was compared in Figure 2D. Single mutants I936A and L994E had selectivity ratios of around 1.5 and 3, respectively. Interestingly, the helix 2-3 single mutant (L994E) reduced LD2 binding by about twice over the helix 1-4 single mutant (I936A). Most notably, the double mutant, L994E/I936E had a selectivity ratio of 18. The decrease in R_eq_ from WT to the mutants validated these constructs as controls for testing the binding specificity of fragments to the FAT domain.

### 2.5. Backbone Resonance Assignments for Human FAT Domain

**Figure 3:**
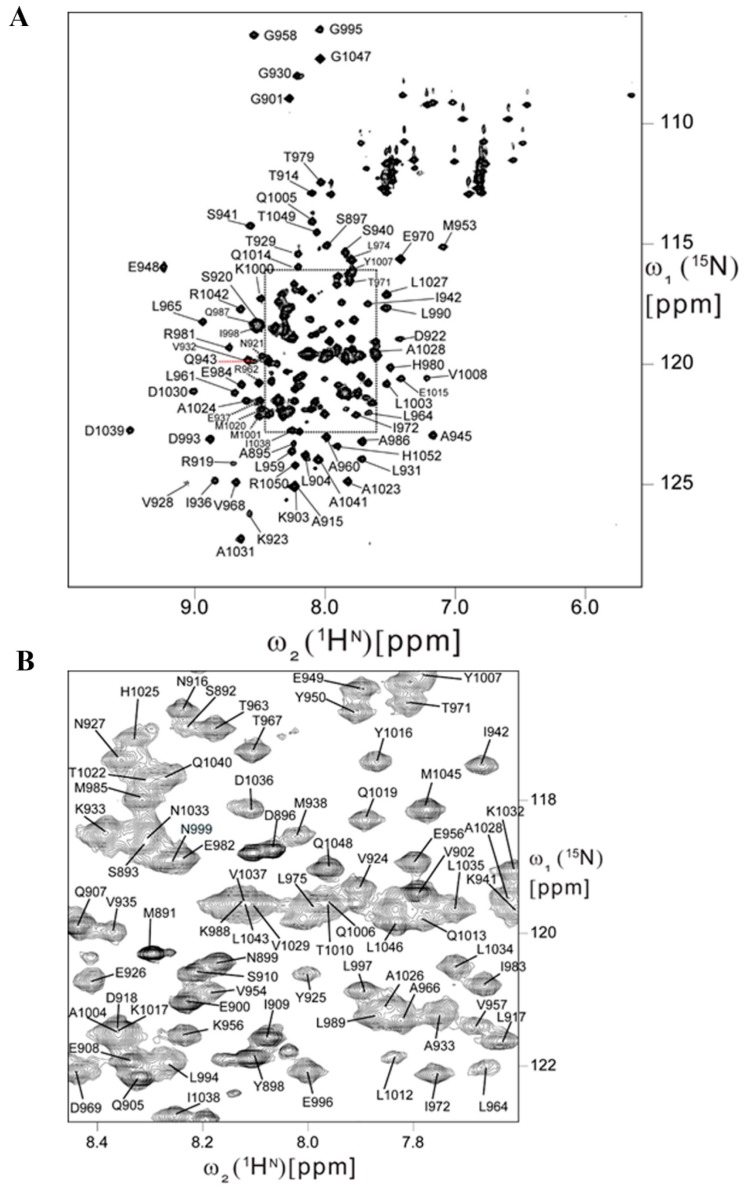
Backbone resonance assignments of human FAT. (**A**) 2D [^15^N,^1^H] HSQC spectrum of human FAT-892 recorded at 750 MHz ^1^H resonance frequency and 37 °C in about 1.5 h. Resonance assignments are indicated using the one-letter amino acid code and the numbering of the full-length protein. (**B**) Central spectral region of the 2D [^15^N,^1^H] HSQC spectrum of the FAT domain.

HSQC NMR is an excellent technique for secondary validation during fragment screening and structure-guided elaboration/linking of a fragment hit. To develop this method, sequence specific backbone resonance assignments (Figure 3) were obtained for human FAT by analyzing triple resonance experiments (3D HNCO, 3D HN(CA)CO, 3D CBCACONH, 3D HNCACB). Overall, sequence specific resonance assignments were obtained for about 90% of the backbone ^15^N, ^1^H^N^, ^13^CO, ^13^C^α^ and ^13^C^β^ nuclei. Backbone NH signals in 2D [^15^N,^1^H] HSQC are exchange broadened beyond detection for residues S889, S978, N991, K1002, T1010, S1011, L1012, K1018, L1021, and K1044, indicating the presence of internal motional modes with correlation times in the µs to ms range. The location of α-helices based on chemical shift index (CSI) and characteristic sequential and medium range NOEs are in very good agreement with X-ray crystal structure [37] (PDB ID: 1K05). Minor differences were observed solely for the lengths of the helices: Helix I: K923–Q943 (X-ray structure PDB ID-1K04: K923–I942), Helix II: P947–I972 (P947–L974), Helix III: T979–Q1006 (A977–Q1006), and Helix IV: Q1013–M1045 (Q1013–M1045). The scarcity of sequential and absence of medium range NOEs reveals that the N-terminal polypeptide segment comprising residues S892–D922 is flexibly disordered in solution. Importantly, the construct used for this study has an additional 22 residues at the N-terminus. Chemical shifts were deposited in the BioMagResBank with accession number 28012. Our backbone resonance assignments represent the first for the human FAT domain.

### 2.6. Feasibility of HSQC-NMR Assay

**Figure 4:**
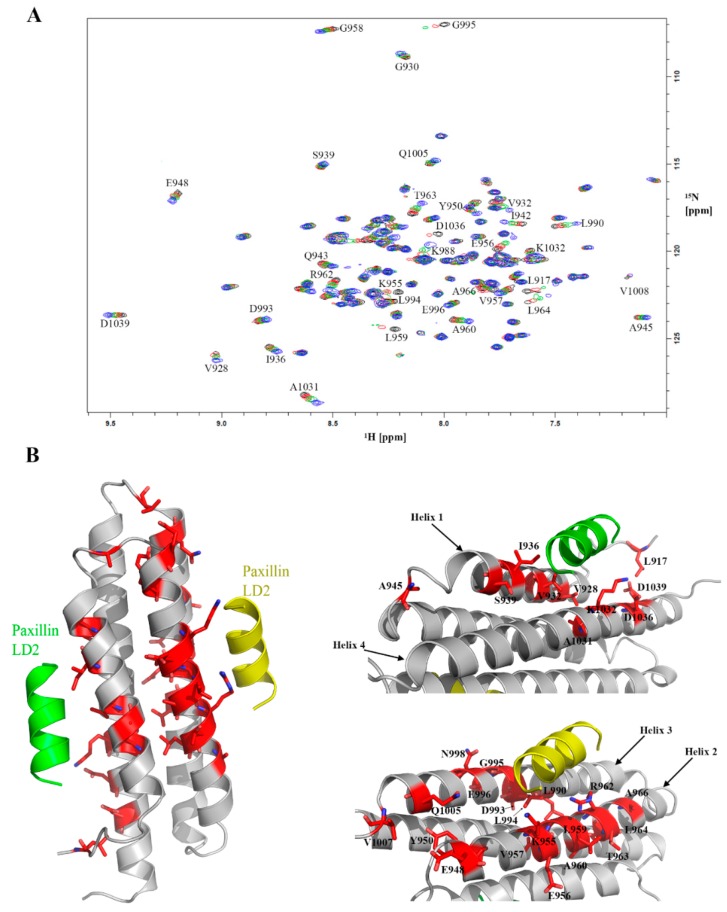
Development of 2D heteronuclear single quantum coherence nuclear magnetic resonance (HSQC-NMR) assay. (**A**) Overlay of 2D HSQC spectra acquired with a 100 µM FAT sample in the presence of varying concentrations of LD2. Two-dimensional HSQC signals represented in blue, green, red, and black are obtained in the presence of 125 µM, 62.5 µM, 31.3 µM of LD2 and 2% DMSO, respectively. Residues displaying notable chemical shift perturbations (CSPs) are indicated with the one letter amino acid code and the numbering of the full-length protein. (**B**) Molecular model (PDB ID: 1OW8) of the FAT-LD2 binding complex with the CSPs from the LD2 titration highlighted in red. A zoomed in view of both the helix 1-4 and helix 2-3 binding sites with proper amino acid labels indicating that the CSPs line up with the model for LD2 binding.

HSQC chemical shift perturbations (CSPs) studies were performed where LD2 peptide was titrated from 500 µM to 500 nM (Figure 4A). The HSQC spectra of the titration demonstrated global CSPs at the higher concentrations indicating binding and conformational changes associated with the large interaction surface between LD2 and FAT. When examining lower concentrations of LD2, we noticed that the CSPs were less ubiquitous. Mapping of these CSPs to the X-ray crystal structure of the LD2-FAT complex (PDB ID: 1OW8) showed significant CSPs at key residues on both binding interfaces of the FAT domain (Figure 4B). The mapping demonstrated that key residues in the helix 2-3 binding interaction (such as L959, K955, E956) exhibit the largest CSPs. In addition, significant CSPs were still observed at the helix 1-4 binding interface (T963, I936, D1036). It is worth noting that the helix 2-3 binding site had more CSPs than the helix 1-4 binding site. However, due to global CSPs at higher concentrations of LD2 and large conformational changes, a binding isotherm could not be derived for each LD2 binding site. In all, these studies are in neat agreement with the residue backbone assignments and thus validated the HSQC NMR assay as a secondary assay for fragment screening.

### 2.7. Pilot SPR Screening and Hit Validation for A Small Fragment Library

**Figure 5:**
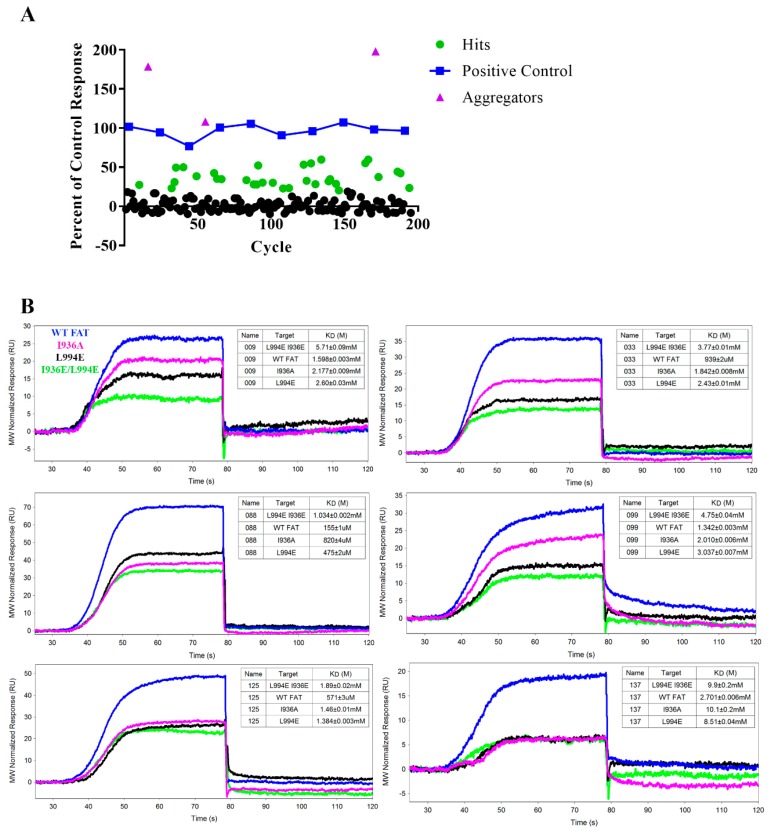
Pilot SPR fragment screen (**A.**) Hit plot displaying results of the primary SPR fragment screen with WT AviTag FAT compared to a control compound found through a fluorescence polarization assay. Hits were characterized as having 20% of the control response. Three hits were above the 100% control response, but analysis of the binding curves showed curves that they didn’t reach equilibrium indicating aggregation and were ruled out as hits. The primary screen produced a total of 32 initial hits. (**B**) Six representative SPR sensorgrams of fragment hits with binding to WT FAT (blue), single mutants I936A (pink), L994E (black), and double mutant I936E/L994E (green). Each binding curve displays their respective K_D_. Careful visual inspection of the binding curves allowed for a final curated hit count of 19 fragments.

A small (189 compound) but molecularly diverse (principal component analysis) fragment library was acquired from Enamine for pilot fragment screening (Enamine Essential Fragment Library Set). Each fragment was screened for binding to WT AviTag FAT on SPR at 1 mM utilizing the OneStep method and compared to a positive control compound (previously identified in a HTS) that was periodically injected over the duration of the assay to verify surface integrity (Figure 5A and Figure S1) [36]. Buffer was flowed over the chip for 1 min after each injection, as the weak binding nature and quick dissociation of the ligands allowed for simple regeneration. The data was reported as a percent of the control response with 20% of the control response being the threshold for hit identification. From this initial screen, 32 fragments met the hit threshold and a further three fragments demonstrated percent responses higher than 100. From visual inspection of the binding curves, all three compounds that were above the positive control had binding kinetics that did not reach binding equilibrium, indicating non-specific binding effects commonly seen in aggregators. Twenty-nine of the 32 fragment hits were available for further binding validation by SPR using WT FAT and each of the mutant FAT constructs. All kinetic data as well as the WT/mutant selectivity ratio are shown in Table 1 and Figure 5B shows 6 representative curves from the pilot screen. Compound 088 had the highest affinity to WT with a K_D_ of 155 ± 1 µM, but compound 047 had the best WT/double mutant selectivity ratio at 5.18. Interestingly, some compounds exhibited stronger binding affinities to some mutants over WT, such as compounds 063, 059, 081, 092, 121, and 141. Compounds 131, 088, and 181 had the highest ligand efficiencies at 0.49, 0.44, and 0.40, respectively, providing decent starting points in the drug development process. Ten compounds had curves that either did not reach equilibrium, had excessively low on/off rates, or did not reach baseline after dissociation. This classification indicated either non-specific binding interactions or aggregation and were thus ruled out as hits, leaving the final curated hit count after SPR at 19.

### 2.8. Validation of Hits Using HSQC-NMR

**Figure 6:**
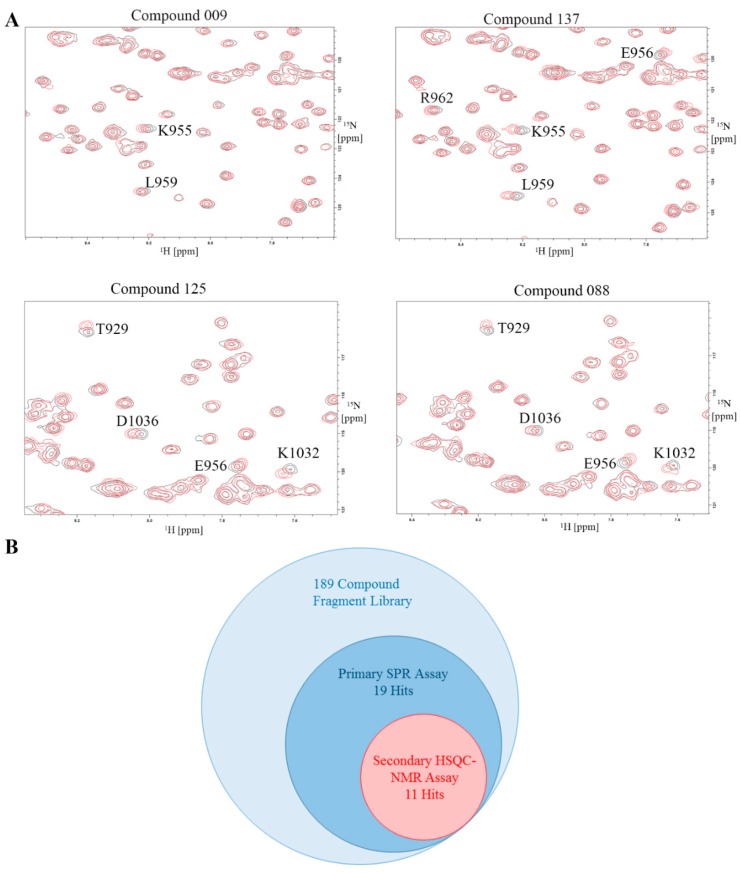
Validation of hits from SPR utilizing 2D HSQC-NMR. (**A**) Representative HSQC overlays displaying the CSPs of four hits. Compounds 009 and 137 demonstrate multiple CSPs in the helix 2-3 binding site. Compounds 125 and 088 demonstrate a mix of helix 2-3 and 1-4 binding. (**B**) Venn diagram detailing the number of hits after each screen. The initial library was 189 compounds, with 19 curated hits after SPR, and a final 11 hits after secondary screening by 2D HSQC-NMR.

The 29 available compounds, which includes the 19 curated SPR hits, were run at 1 mM through HSQC-NMR as a secondary assay to validate the hit identification from SPR. Four representative HSQC-NMR overlays are presented in Figure 6. The 10 compounds removed from the initial hit list that exhibited non-specific binding in SPR had no or very minimal CSPs shown on the HSQC spectra. Eight additional compounds from the curated list also showed no CSPs, leaving a total of 11 final hits after the secondary assay. All the hits had the largest CSPs on either residues L959, K955, or E956, all of which are on the helix 2-3 binding site. Two compounds (compounds 125 and 088) also showed CSPs on helix 1-4 residues T929 and D1036, indicating dual site binding properties.

## 3. Discussion

FAK is critical in cancer development due to its function as a central component to multiple oncogenic signaling pathways [4,5,6,7]. This article reports an alternative approach in the discovery of FAK inhibitors, specifically in targeting the FAT-Paxillin interaction. The use of fragment-based drug discovery to gather and analyze hit compounds has increased in prevalence over the past 25 years [38]. By utilizing this different approach in drug discovery, the FAT-Paxillin binding interface can be explored through improved sampling of chemical space by these smaller molecules. Fragment hits can then serve as building blocks for possible lead compounds.

To develop a SPR-based FAT FBDD screening platform, we first designed an AviTag construct of FAT and adopted the OneStep injection method. The comparison of OneStep to FCI validated the OneStep injection, with a K_D_ around the same range as FCI. This switch to OneStep allowed us to conserve reagents and time while providing a relatively accurate estimate for K_D_ compared to a fixed concentration injection. We then optimized buffer conditions, and interestingly, Buffer 3 containing Triton X-100 demonstrated non-ideal binding kinetics and non-specific binding with no LD2 present. We hypothesize that the structure of Triton X-100 is conducive to non-specific interactions with our immobilized AviTag FAT construct, making Tween-20 the better detergent to use. Intriguingly, Buffer 6 containing 5% DMSO had the highest binding response of all buffers tested. We speculate the higher DMSO concentration allows for greater LD2 solubility promoting more binding interactions. Following the mutant study with LD2, results showed that LD2 has a higher selectivity ratio for L994E (3) than I936A (1.5) indicating preferential binding of LD2 to the 2-3 binding site over the helix 1-4 site.

To further validate any fragment hit from SPR, a secondary HSQC-NMR assay was developed, this provided an orthogonal fragment screening platform for FAT drug discovery. In the LD2 titration on our HSQC-NMR assay, the helix 2-3 binding site has almost twice the amount of CSPs over the helix 1-4 binding site further strengthening the argument that the 2-3 site has a higher affinity for LD2. The backbone resonance assignments acquired are also the first reported on human FAK. The FAT construct used had an extra 30 amino acids on the N-terminal from previously determined avian FAT constructs used for resonance assignments, making it the largest FAT construct to have backbone assignments reported. NMR also demonstrated that FAT forms a dimer in solution consistent with a previously reported crystal structure (PDB ID: 1K04) [37]. The dimer adopts a helix-swapped conformation where helix 1 is exchanged between two different FAT molecules. However, going off the crystal structure, each unit of the dimer retains the same tertiary structure as monomeric FAT, suggesting dimer formation has minimal impact on binding.

The results of our fragment library screen demonstrate the sensitivity of both assays. Due to their small inherent size, fragments typically have weaker binding affinities. As shown in Table 1, all hits have a K_D_ > 100 µM with a good majority being in the low mM range. These low affinity fragments still produce a detectable response on SPR with binding curves capable of being fit through a kinetic model. Through HSQC-NMR, CSPs of low affinity compounds are still measurable. Such high sensitivity assays are required to properly detect fragment hits. Remarkably, a couple fragment hits exhibit similar binding trends to LD2, where they have a higher selectivity ratio for L994E over I936A, indicating preferential binding to the 2-3 site, such as compounds 033 and compounds 009 shown in Figure 5B. In Figure 6A we also note that compounds 033 and 009 display strong CSPs mapped to the 2-3 binding site, notably at L959 and K955, while having very limited CSPs on the helix 1-4 site. However, not all compounds exhibit the strong preference for only the helix 2-3 site in NMR. Compound 125 shown in Figure 6 shows CSPs at T929 and D1036 on the helix 1-4 site.

Hit selection was based purely on the binding curve to the WT, however selectivity ratio and binding affinity to the mutants are key in prioritization of the fragment hits. Certain fragment hits such as compound 168 demonstrate higher selectivity ratios to the single mutant than to WT, perhaps representative of significant conformational changes in the double mutant protein. Other fragments like compound 141 have selectivity ratios less than one for the single mutants, which could suggest allosteric binding to FAT. However, due to the weak estimated binding affinity of this compound (>8 mM), it was difficult to observe CSPs in HSQC NMR and thus could not identify an alternative binding site. While allosteric binding could be a potential approach to inhibiting the FAT-Paxillin interaction, direct competitors will be prioritized in future studies making selectivity ratio key for compound selection and iterative drug discovery.

Notably, all fragment binders showed CSPs in the same region of the 2-3 binding site on their HSQC maps. Residues L959, E956, and K955 consistently show CSPs on fragment hits, however other residues on a different region of the helix 2-3 site such as L964 and A966 show none. Our NMR assay can be used to perform a second site screen where we search for binders that produce CSPs on this secondary region within the helix 2-3 site. If such compounds are found, linking of fragment hits from both regions in the helix 2-3 site could produce a more potent inhibitor.

To conclude, we were able to successfully develop two orthogonal fragment screening assays in SPR and NMR and screened a small fragment library as a pilot, representing the first FBDD screen against the FAT domain. Furthermore, we obtained nearly complete backbone resonance assignments for the human FAT domain encompassing residues S892-H1052 from full length FAK. The pilot screening of this fragment library allows for a great starting point in the FBDD pipeline. Out of the 189 fragments screened, a final 11 compounds are suitable starting points for further optimization through medicinal chemistry. Our next steps will be a fragment screen with a larger fragment library to potentially identify new fragments with alternative chemotypes and different binding sites. Also, we intend to grow out the fragments by SAR to enhance the binding affinity to FAT. Furthermore, we will explore X-ray crystallography studies to identify the three-dimensional conformation of our fragment hits in the binding pocket. Using HSQC-NMR, we can perform second site screening of the helix 2-3 binding site to find linking partners to our current fragment hits [39]. The near 6% hit rate presented in this paper further validates the sensitivity of both assays and the potential of FBDD to probe a complex binding interface. These results are a major step forward in the search for a first-in-class non-catalytic FAK inhibitor that may show enhanced efficacy compared to traditional ATP-competitive kinase inhibitors.

## 4. Materials and Methods

### 4.1. Construct Design, Cloning, Expression, and Purification of Biotinylated FAT

Oligonucleotides (forward 5′-GCTACCATGGGCAGCAGCCATCATCATCATCATCACAG-CAGCGGCCTGGTGCCGCGCGGCAGCGGCCTGAACGACATCTTCGAGGCTCAGAAAATCGAATGGCACGAACATATGCTCG-3′ and reverse 5′-CGAGCATATGTTCGTGCCATTC-GATTTTCTGAGCCTCGAAGATGTCGTTCAGGCCGCTGCCGCGCGGCACCAGGCCGCTGCTGTGATGATGATGATGATGGCTGCTGCCCATGGTAGC-3′) containing the AviTag sequence (GLNDIFEAQKIQWHE) [34], a hexahistdine tag, and a thrombin site in-between both tags were designed and ordered through ThermoFisher (Waltham, MA, USA). The oligonucleotides were solubilized at 1 µg/µL in 10 mM Tris pH 8.0, 1 mM EDTA (TE Buffer). 2 µg of each oligonucleotide were annealed together in a thermocycler in TE with 50 mM NaCl using the following protocol: Heat to 94 °C for 2 min, then gradually cool by −2 °C every 1 min until 4 °C. The annealed double stranded oligonucleotide was cloned into a pET15b FAT construct, residues S892-H1052, using restriction sites for NcoI and NdeI. The AviTag FAT construct was expressed in BL21(DE3) and purified using Ni-NTA resin. Purified AviTag FAT was buffer exchanged into biotinylation buffer (10 mM Tris pH 8.0, 500 mM potassium glutamate) and biotinylated using BirA biotin ligase purchased from Avidity (Aurora, CO, USA) following the manufacturer protocol. Biotinylation efficiency was tested through western blot using biotin primary antibody and comparing to biotinylated AviTag Maltose Binding Protein purchased from Avidity. Biotinylated protein was gel filtered into 20 mM Tris pH 8.0 and 100 mM NaCl to remove BirA using a HiLoad Superdex 75 pg 26/60 column at a flow rate of 0.5 mL/min.

### 4.2. Surface Plasmon Resonance (SPR)

SPR binding studies were performed on a ForteBio Pioneer FE (Fragment Edition) SPR system utilizing a SADH Streptavidin-coated Dextran Hydrogel biosensor (ForteBio, San Jose, CA, USA). SPR running buffer was optimized and the final buffer chosen was 100 mM Tris-HCl, 200 mM NaCl, 0.05% Tween-20 (Buffer 6). Each biotinylated Avitag-FAT construct to be tested was diluted in Buffer 6 and injected at 10 µL/min to achieve approximately 1000 RU of immobilized protein for peptide studies during optimization and 7000 RU for fragment screening. Channel 2 was always left empty as the reference control. Immobilization levels were decided based on the following equation:

(1) $$R_{max}=R_{protein}\times \left(\frac{Mr_{analyte}}{Mr_{protein}}\right)\times V_{protein}$$

where *R_max_* is the highest possible SPR response given by an analyte binding to an immobilized protein. *R_protein_* is the response change attributed to the immobilization of the ligand. *M_r_* are the molecular masses of analyte and protein. *V_protein_* is the number of binding sites per protein available for analyte binding. Through this equation, by aiming for an *R_max_* of around 150 RU, the above immobilization levels were derived for peptide and fragment studies. All samples tested were diluted in Buffer 6 injected using the OneStep gradient injection method at a flow rate of 150 µL/min. 3% sucrose was utilized as a bulk standard control for OneStep injection and a DMSO calibration curve was performed using a concentration range of 3.5% to 6.5% DMSO. Fragments were tested for solubility at 1 mM in the above buffer by centrifugation. SPR data was processed through Qdat software (ForteBio) allowing for the normalization of the baseline prior to injection, alignment of the channels, subtraction of the reference channel, and blank subtraction. Kinetic data were fitted to a pseudo-first order 1:1 interaction binding model to calculate *K_D_*. Mutant selectivity ratios were calculated by the following equation, where *R_eq_* represents level of response achieved at binding equilibrium.

(2) $$Selectivity\;ratio=\frac{WT\;R_{eq}}{Mutant\;R_{eq}}$$

Ligand efficiency (LE) of binding fragments was calculated by the following equation, where R is the ideal gas constant, T is the analysis temperature in Kelvin, HAC is the integer value of non-hydrogen (heavy) atoms in the molecule, and *K_D_* is the equilibrium dissociation constant.

(3) $$LE=\left(-2.303\times \left(\frac{RT}{HAC}\right)\right)\times logK_{D}$$

### 4.3. Purification of Isotopically Labeled Protein

[U−^15^N]-labeled FAT, encompassing residues S892-H1052, is expressed using minimal media with enhanced M9 salts: (6.8 g/L Na_2_HPO_4_, 3 g/L KH_2_PO_4_, 0.5 g/L NaCl, 1 g/L ^15^NH_4_Cl, 2 mM MgSO_4_, 100 µM CaCl_2_, 0.4% glucose, 500 µM Thiamin, 0.5× MEM vitamins and 10 µM of FeCl_3_∙6H_2_O, CuSO_4_∙5H_2_O, MnSO_4_·H_2_O, and ZnSO_4_·7H_2_O). Purified ^15^N labeled FAT was buffered exchanged into thrombin cleavage buffer (50 mM Tris pH 8.0, 10 mM CaCl_2_). Buffer exchanged FAT was incubated with 3 mL of Thrombin beads (Sigma Aldrich, St. Louis, MO, USA, Catalog No: RECOMT) at a concentration of 1 mg/mL. The thrombin reaction proceeds shaking for 6 h at room temperature. Thrombin beads were removed through centrifugation at 500 g for 2 min. Supernatant was collected and incubated with Ni-NTA beads to remove uncleaved protein and HisTag remnants. Cleaved protein was gel filtered through a HiLoad Superdex 75 pg 26/60 column into NMR buffer (100 mM Tris pH 8.0, 100 mM NaCl, 2 mM DTT, 50 µM DSS, 0.02% sodium azide and 5% D_2_O). [U−^15^N,^13^C]-labeled 19 kDa FAT-892 (comprising residues Ser892 to His1052 of full-length protein) was prepared similarly at 0.5 mM concentration in 90% H_2_O/10% D_2_O, 100 mM Tris pH 8.0, 100 mM NaCl, 2 mM DTT, 50 µM DSS and 0.02 % sodium azide. An isotropic overall rotational correlation time of about ~16.5 ns was inferred from average backbone ^15^N spin relaxation times [40], indicating that the protein is dimeric in solution. This is consistent with one X-ray crystal structure of human FAT-892 (PDB ID-1K04) featuring a helix-swapped dimer [37].

### 4.4. FAT Backbone Resonance Assignments

NMR experiments needed for backbone assignments were acquired at 37 °C on Agilent (Agilent Technologies, Inc., Santa Clara, CA, USA) DD2 600 MHz and Varian INOVA 750 MHz spectrometers (Varian, Inc., Palo Alto, CA, USA) equipped with cryogenic and room temperature ^1^H{^15^N,^13^C} probes, respectively. NMR spectra for backbone and side-chain resonance assignments were acquired on a ^13^C, ^15^N FAT sample that begins at residue 892 with a GSHM overhang after thrombin cleavage. Spectra were processed using the program PROSA 6.6 [41] and analyzed using the programs XEASY [42] and CARA [43]. ^1^H chemical shifts were referenced relative to 4,4-dimethyl-4-silapentane-1-sulfonic acid (DSS), and ^13^C and ^15^N chemical shifts were referenced indirectly based on radio-frequency carrier frequencies. Sequence specific backbone (Figure 3) and ^13^C^β^ resonance assignments were obtained from 3D HNCO, 3D HN(CA)CO, 3D CBCACONH, and 3D HNCACB. The assignments were confirmed based on sequential NOEs observed in 3D [H]-NOESY-[NH/CH] (τ_mix_ = 70 ms). Side-chain ^1^H^α^, ^1^H^β^ assignments were obtained from 3D HBHA(CO)NH. The location of α-helices was identified based on a consensus chemical shift index (CSI) [44] and patterns of sequential and medium range NOEs. In total, 13 NMR experiments were acquired for resonance assignments, listed in Table S1.

### 4.5. D HSQC-NMR

Two-dimensional HSQC-NMR samples are all prepared at a ^15^N FAT concentration of 100 µM. Fragments were screened at a concentration of 1 mM with a DMSO concentration of 1%. LD2 titration reported in Figure 3 were carried out in buffer containing 2% DMSO, the titration ranging from 500 µM down to 250 nM of LD2. 2D TROSY based HSQC were collected on a Bruker Avance III-HD console equipped with a 5mm TCI Prodigy cryoprobe (Bruker Corp., Billerica, MA) operating at resonance frequencies of 600.133 and 60.817 MHz for ^1^H and ^15^N, respectively. Each 2D experiment was collected with spectral widths of 14.03 and 30.0 ppm, 2048 and 256 points respectively, and 16 scans per FID averages [45,46,47,48,49]. All spectra were processed using the Bruker TopSpin software package (Bruker Corp., Billerica, MA, USA).

### 4.6. Site-Directed Mutagenesis of AviTag FAT

Mutation primers for L994E (5’-CTTGTTGATGAGCTCACCCTCGTCAGAGTTCAATAGC-TTC-3’ and 5’-GAAGCTATTGAACTCTGACGAGGGTGAGCTCATCAACAAG-3’), I936A (5’-GATTTTACTGGACATCTCGGCGACAGCTTTCACCAGGCCC-3’ and 5’-GGGCCTGG-TGAAAGCTGTCGCCGAGATGTCCAGTAAAATC-3’), and I936E (5’-GGATTTTACTGGA-CATCTCCTCGACAGCTTTCACCAGGCCC-3’ and 5’-GGGCCT-GGTGAAAGCTGTCGAG-GAGATGTCCAGTAAAATCC-3’) were designed through Agilent Technologies (Santa Clara, CA, USA). The Agilent QuikChange Lightning kit was used to complete the mutations. Mutations were verified through sequencing and each new construct was purified and biotinylated in the same way as the wild type construct.

## Figures and Tables

**Table 1:** Focal Adhesion Kinase (FAK) FAT binding affinity, WT/Mutant selectivity ratio, ligand efficiency, and HSQC-NMR validation for fragment hits on SPR screen.

Compound	Wild Type K_D_ ± SE (µM)	I936A Single Mutant K_D_ ± SE (µM)	L994E Single Mutant K_D_ ± SE (µM)	I936E/L994E Double Mutant K_D_ ± SE (µM)	I936A Selectivity Ratio	L994E Selectivity Ratio	I936E/L994E Selectivity Ratio	Ligand Efficiency (WT)	Validated by HSQC NMR
009	1598 ± 3	2177 ± 9	2600 ± 30	5710 ± 90	1.27	1.64	2.95	0.34	X
030	ND	ND	ND	ND	2.09	2.27	1.35	ND	
032	ND	ND	ND	ND	0.92	1.55	1.37	ND	
033	939 ± 2	1842 ± 8	2430 ± 10	3770 ± 10	1.57	2.11	2.62	0.31	
038	ND	ND	ND	ND	1.01	0.94	0.83	ND	
047	1780 ± 30	2400 ± 70	3000 ± 200	10,000 ± 2000	1.34	1.97	5.18	0.26	
059	2900 ± 10	2215 ± 9	857 ± 2	1286 ± 4	0.86	0.76	1.56	0.26	X
061	ND	ND	ND	ND	1.65	1.35	0.92	ND	X
063	884 ± 3	671 ± 3	418 ± 1	2088 ± 5	0.90	0.94	1.95	0.32	X
081	1815 ± 9	4310 ± 40	483 ± 2	1041 ± 3	1.97	1.46	1.37	0.28	
085	ND	ND	ND	ND	1.76	1.53	1.42	ND	X
087	3070 ± 20	3310 ± 90	7100 ± 300	ND	1.22	2.95	ND	0.39	
088	155 ± 1	820 ± 4	475 ± 2	1034 ± 2	1.82	1.59	2.06	0.44	X
092	3542 ± 9	3810 ± 20	3450 ± 8	2407 ± 4	1.10	1.23	1.04	0.26	
099	1342 ± 3	2010 ± 6	3037 ± 7	4750 ± 40	1.33	2.10	2.62	0.31	X
105	ND	ND	ND	ND	ND	ND	ND	ND	
109	896 ± 3	6300 ± 100	4230 ± 10	ND	4.02	3.16	2.07	0.30	X
119	3200 ± 10	3890 ± 60	3920 ± 10	5490 ± 20	1.30	1.38	1.51	0.27	
121	5030 ± 30	8900 ± 100	3055 ± 5	8240 ± 20	1.62	1.79	1.70	0.28	
125	571 ± 3	1460 ± 10	1384 ± 3	1890 ± 20	1.75	1.82	2.15	0.35	X
131	292 ± 2	1770 ± 10	865 ± 2	1121 ± 2	2.33	1.71	1.79	0.49	X
136	ND	ND	ND	ND	0.64	0.36	0.53	ND	
137	2701 ± 6	10,100 ± 200	8510 ± 40	9900 ± 200	2.96	2.96	3.27	0.30	X
141	8120 ± 20	6610 ± 60	3088 ± 6	10,140 ± 20	0.89	0.99	1.89	0.22	
143	ND	ND	ND	ND	2.68	2.68	1.62	ND	
162	ND	ND	ND	ND	ND	ND	ND	ND	
168	1830 ± 10	6200 ± 200	4490 ± 40	2910 ± 30	3.07	2.43	1.48	0.32	
181	709 ± 1	1682 ± 3	1240 ± 2	1029 ± 2	1.64	1.86	1.28	0.40	
183	ND	ND	ND	ND	ND	ND	1.37	ND	

Abbreviations: ND—Binding affinities not determinable due to irregular kinetics or lack of consistent binding.

## Supplementary Materials

The following are available online. Table S1. NMR experiments measured for human FAT residue backbone resonance assignments. Figure S1. Structure of control compound for SPR screening.
