# Supplementary material for: Development of a Fragment-Based Screening Assay for the Focal Adhesion Targeting Domain Using SPR and NMR
Source: Molecules. 2019 Sep 14;24(18):3352. doi: 10.3390/molecules24183352 (PMC6766811; doi:10.3390/molecules24183352)
Supplement: Supplementary file 1 [file molecules-24-03352-s001.pdf]

Table S1. NMR experiments measured for human FAT backbone resonance assignments.

Experiment Number	Experiment Type	Spectrometer (MHz)
1	1D 1H	600
2	1D 1H decoupled	600
3	1D [^{15}N,^{1}H] HSQC T1	600
4	1D [^{15}N,^{1}H] HSQC T2	600
5	2D [^{15}N,^{1}H] HSQC & TROSY	600 & 750
6	2D [^{13}C,^{1}H] *ct* HSQC (aliphatic)	600 & 750
7	2D [^{13}C,^{1}H] *ct* HSQC (aromatic)	600 & 750
8	3D HNCO	600
9	3D HN(CA)CO	600
10	3D HNCACB	600
11	3D CBCACONH	600
12	3D HBHACONH	600
13	3D [^{1}H]-NOESY-[NH/CH]	600

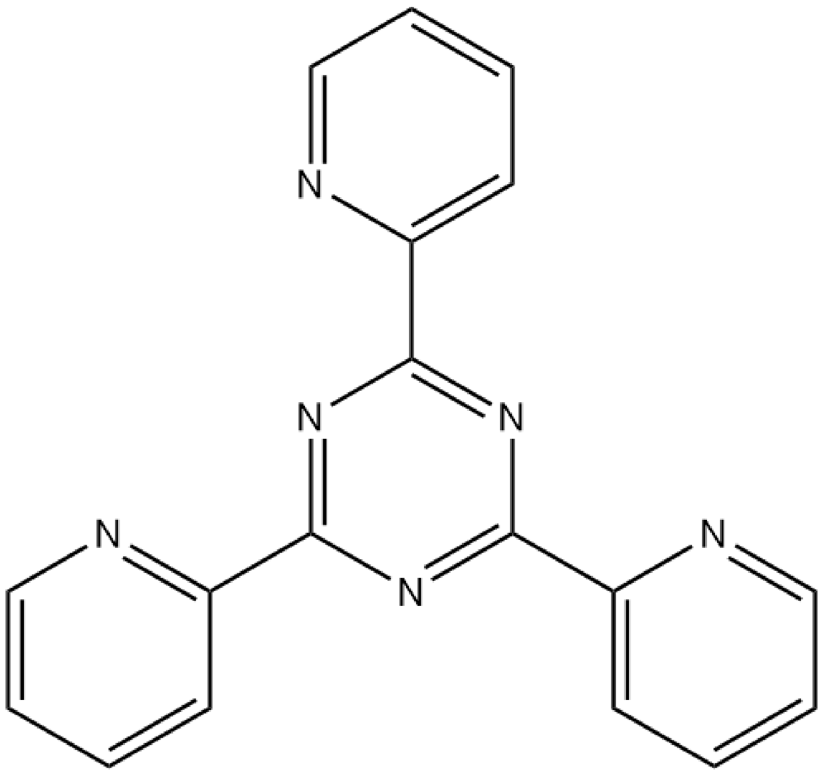

Figure S1. Structure of control compound for SPR screening (2,4,6-tri(pyridin-2-yl)-1,3,5-triazine).
